# Dropout and compliance to physical exercise in menopausal osteopenic women: the European “happy bones” project

**DOI:** 10.3389/fspor.2023.1221409

**Published:** 2023-06-27

**Authors:** Elisa Grazioli, Claudia Cerulli, Eliana Tranchita, Elisa Moretti, Caterina Mauri, Marianna Broccatelli, Simona De Bellonia, Beatrice-Aurelia Abalașei, Iulian-Marius Dumitru, Cristina-Elena Moraru, Radu-Mihai Iacob, Sergi Blancafort-Alias, Aimar Intxaurrondo González, Àlex Domingo Riau, Albert Giménez i Sanz, Graciela Inness Ramos, Haci Ahmet Pekel, Mustafa Altunsoy, Secil Ozkan, Asiye Ugras Dikmen, Massimo Sacchetti, Attilio Parisi

**Affiliations:** ^1^ Department of Movement, Human and Health Sciences, Rome, Italy; ^2^ Faculty of Physical Education and Sports, University “Alexandru Ioan Cuza” of Iași, Iași, Romania; ^3^ Fundació Salut I Envelliment UAB, Barcelona, Spain; ^4^ Fundació Claror C/de Sardenya, Barcelona, Spain; ^5^ Department of Public Health, Faculty of Sport Sciences, Gazi University, Yenimahalle/Ankara, Türkiye; ^6^ Department of Public Health, Faculty of Medicine, Gazi University, Yenimahalle/Ankara, Türkiye

**Keywords:** exercise, workplace, sport center, women, compliance, health, bone

## Abstract

**Introduction:**

Decline in muscle mass and bone density seem to be two of the most disabling side effects of menopause that negatively affect women's quality of life. Promoting physical activity protocols in the workplace can represent a focal point in the prevention and management of several diseases. The study aims to evaluate the compliance and drop-out of menopausal osteopenic women engaged in combined training performed inside and outside the workplace. Strength and balance were analyzed to evaluate the effect of this protocol on osteoporosis prevention and the risk of falling.

**Methods:**

73 menopausal women were enrolled in 5 European countries. They performed 72 lessons of a combined training proposed in the working place (IW) or sport center (SC).

**Results:**

Out of the total 39 women enrolled in the IW, 12.8% had to leave the program, while out of the 34 women enrolled in SC, 41.2% did not complete the training. According to the compliance results, 47% of women that completed the trained IW and 85% in the SC recorded high compliance (*p* = 0.019). Moreover, the strength of the lower limbs (*p* < 0.001) and static balance (*p* = 0.001) significantly improved in the whole group.

**Discussion:**

In conclusion, proposing well-structured training in the workplace for menopausal women seems to reduce drop-out. Strength and balance results suggest its positive impact on bone health and risk of falls, despite where it is performed.

## 1. Introduction

Menopause is a unique event in women's life that occurs around the age of 50 and it is a stage that all women would experience (1, 2). It is characterized by the progressive decline of female hormones, estrogen, and progesterone, culminating in a total shutdown of the ovaries (3). This condition can lead to several side effects, such as an increase in fatigue, headache, and mood swing, that can negatively affect the Quality of Life (QOL)(4). Moreover, physical performance declines with aging and studies reported that this decline differs by sex, with women showing a more rapid decline than men during middle age (5, 6). This sex difference may be due to the hormonal changes that occur during the menopausal years (7, 8). For these reasons, menopausal women are particularly exposed to the risk of osteopenia and osteoporosis, and fragility fracture (9). It is known that in 2025 there will be an increase in the risk of falls, which will most likely lead to fractures in the case of osteopenia/osteoporosis (10). According to the International Osteoporosis Foundation (IOF), to avoid premature bone loss, adults should implement a nutritious diet with adequate calcium intake, maintain healthy body weight and participate in regular weight-bearing activity (8). Well-structured exercise protocols can make a series of adaptations which, in addition to having a positive effect on bone health, have a direct influence on the prevention of falls, improving the strength of the lower limbs and balance. Moreover, Physical Activity (PA) has been shown to enhance physical functions and QOL among menopausal women, improving psychosomatic well-being and physiological parameters (11, 12). It is therefore essential that menopausal women practice PA regularly, both to preserve bone health and to maintain strength and balance. Despite all this knowledge, women in general, and those in menopause in particular, do not reach the minimum amount of PA recommended (13). This highlights the urgent need to find a new strategy to implement adherence to the Moderate Vigorous PA as well as compliance with a well-tailored protocol of PA. The term adherence indicates the number of minutes engaged in Moderate-Vigorous PA in unsupervised and unstructured environments, while the term compliance indicates the frequency of participation in terms of the number of sessions attended in a supervised intervention and it is often analyzed with the addition of the drop-out rate (14). Currently, a real classification of the rate of compliance with the PA program has not yet been defined, but participation that is around 80% can be considered a satisfactory threshold (15). As it is known, the workplace represents an environment in which individuals spend a high number of hours and covers a wide age group with various risk factors, and it is often the work itself that leads individuals to high levels of a sedentary lifestyle, spending many hours sitting (16). In addition, lack of time and lack of transportation are referred to be the primary cause of physical inactivity in people (17). In this framework, the World Health Organization (WHO) Comprehensive Worker Health Action Plan (18) establishes, among other things, that the workplace should provide resources for the personal health of workers and that it should be the first intervention outpost to promote and implement a healthy lifestyle. Indeed, as it was reported in the literature, structured and supervised physical exercise interventions carried out in the workplace, with a minimum duration of four months and with at least two weekly sessions, allow a significant decrease in Body Mass Index (BMI), an increase in muscle mass (19, 20), and an improvement in systolic blood pressure (21). Moreover, in a recent review, it was shown that this type of workplace intervention leads to an increase in Metabolic Equivalent of Task (MET)-mins per week and positively impacts the cardiometabolic health of women of working age in high-income countries (22). In another recent review, it was evidenced that training performed in the workplace can modulate sedentary behavior, reducing the number of prolonged sedentary behaviour greater than 30 min during the whole day (23). According to our knowledge, no studies have so far evaluated the compliance and the effect of a well-tailored protocol proposed in the workplace on menopausal women and compared it to the same protocol performed in sport center outside the working place. Considering these findings, our study aims to evaluate the compliance and drop-out of a well-structured combined training performed inside and outside the workplace in menopausal osteopenic women. Moreover, strength, balance, and QOL will be analyzed to evaluate the effect of this protocol on physical fitness, an indicator of menopausal women's general health needed to prevent osteoporosis and risk of falling (24).

## 2. Materials and methods

### 2.1. Participants

The project was funded by the European Commission (G.A. 613137-EPP-1-2019-1-IT-SPO-SCP—ERASMUS + SPORT). Coordinated by the University of Rome “Foro Italico”, the partnership consists of 5 European partners: the University of Rome “Foro Italico” (Italy), Bulgarian Sports Development Association—BSDA (Bulgaria), Alexandru Ioan Cuza University of Iași—UAIC (Romania), Gazi University—GU (Turkey) and Fundaciò Salut I Envelliment UAB–- FSIE (Spain). Before the start of the study, through the Train the Trainers methodology, the coordinator organized a specific training course for professionals of the exercise, 2 for each partner country, where it was explained the well-tailored protocol for menopausal women as well as all tests performed during the functional and psychological evaluations. Moreover, a dedicated website was created (https://www.happybones.eu/section “intellectual outputs”), where theoretical and practical manuals have been uploaded to trigger the training cascade effect and involve more operators in the sector. Once the Train the Trainers course was completed, a pilot action based on the well-tailored protocol was planned in each partner country, where at least 10 women were enrolled according to the following inclusion criteria: aged between 45 and 65 years, inactive (less than 150 min of PA per week), being in menopause and osteopenic and/or osteoporotic. The osteopenic/osteoporosis status was evaluated through a Dual-energy x-ray absorptiometry (MOC/DEXA) report, the analysis was performed autonomously by the women no more than 12 months before the start of the training. Patients with cardiovascular disease and other complications were excluded. In Italy, Romania, and Turkey participants were enrolled in the Universities through an institutional mail sent by the Human resources office, while in Bulgaria and Spain were distributed informative leaflets in the Sport Centers. The study aimed to evaluate compliance and drop-out with the well-tailored training protocol about the place where it was proposed (workplace or sports center). Moreover, functional (strength, balance, flexibility) and psychological parameters were evaluated to analyze the effect of this protocol on women's general well-being. After signing an informative content, a total of 73 menopausal women aged between 50 and 65 were enrolled. The participants' characteristics are reported in Table 1. No significant differences between countries were reported at baseline. The proposed study protocol was drafted in accordance with the European Unio''s Standards of Good Clinical Practice and the current revision of the Declaration of Helsinki and was approved by the University's Ethics Committee.

**Table 1:** Women's characteristics.

Women's characteristics	Italy *n* = 15 (M ± SD)	Bulgaria *n* = 16 (M ± SD)	Romania *n* = 12 (M ± SD)	Turkey *n* = 18 (M ± SD)	Spain *n* = 12 (M ± SD)	Tot *n* = 73 (M ± SD)
Age (yrs)	56.1 ± 5.4	63.5 ± 2.2	54.8 ± 4	55 ± 3.7	60.5 ± 5.1	58.0 ± 4.1
Weight (Kg)	66 ± 9.9	64.3 ± 11	68.2 ± 5.8	68 ± 11.5	66.8 ± 10.8	66.9 ± 9.5
Height (cm)	164.3 ± 4.7	162.7 ± 9.1	163.5 ± 6.3	159 ± 6.6	157.2 ± 8.1	161.7 ± 6.9
BMI	21	24	20	22	26	23
Group	IW	SC	IW/SC	IW	SC	

n, number; BMI, Body Mass Index; M, mean; SD, standard deviation; Tot, total; IW, in the workplace; SC, Sport Center.

**Figure 1:**
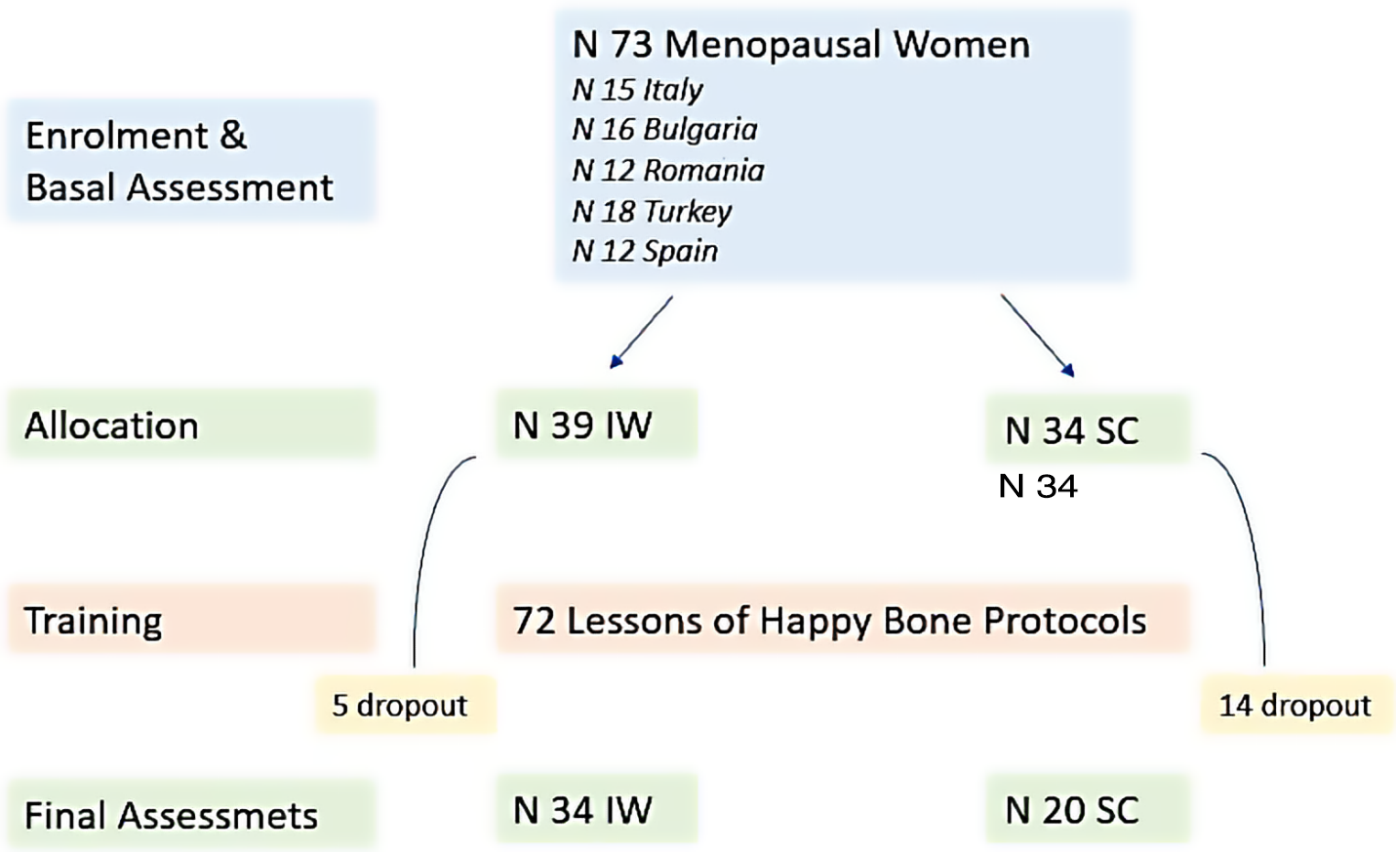
Flowchart of the study design. IW, In the working place; SC, Sport Center; N, number.

From the total of 73 participants, 39 were recruited in the workplace (IW) and they performed the activity in the gym inside the working structure, which is the University involved in the European project (Italy, Romania, Turkey), while 32 women were enrolled among the general population and performed the activity outside the workplace, in Sports Centers (SC) involved in the European project (Bulgaria, Romania, Spain). A detailed flowchart of the study design is reported in Figure 1.

### 2.2. Assessments

Before the start and at the end of the well-tailored training protocol, the physical, functional, and psychological state of all participants in each country was assessed by administering evaluation tests and questionnaires described below:

1Repetition Maximum (RM): to measure the lower limb strength, and was estimated using the Brzycki formula on submaximal loadsMaximum weight = [Weight used in the test/[1,0278—(0,0278 * number of repetitions)] (25). Participants performed three trials with increased loads on each machine used during the protocol: Leg press, Leg extension, Leg curl and Gluteus machine. The formula was carried out using the load lifted for fewer repetitions, less than 8.,

Six Minutes Walking Test: to evaluate the functional capacity. At the end of the test, the effort was indicated by the patient through the Borg scale 0–10 (26).

Handgrip Test: to measure the strength of the handheld grip using a dynamometer (Jamar Plus®, Patterson Medical Ltd.), which is representative of the general strength of the subject (27).

30-second Sit to Stand: to assess the strength of the lower limbs, balance, and risk of falls (28). The participants had to get up and sit properly from a chair and the trainer recorded the number of stands they completed in 30 s.Star Excursion Balance Test: to evaluate the dynamic balance and it is performed with a grid placed on the floor, with 8 lines extending from the center (anterolateral, anterior, anteromedial, medial, posteromedial, posterior, posterolateral, and lateral) (29). The distance reached from the center in each direction is measured in centimetres and subsequently adjusted according to the length of the subject's leg.

Single Leg Stance: to evaluate the static balance. Three trials are carried out for each leg, if 30 s are reached the trial is interrupted (30).

International Physical Activity Questionnaire (IPAQ): this questionnaire is used as a surveillance instrument of Physical Activity among adults and is self-administered. The score obtained allows to convert the activities carried out into MET-min/week (31).

Quality of life Questionnaire (QUALEFFO-41): this psychological questionnaire, developed by the IOF, measures different aspects of the subject's life, such as physical, social, and mental function, pain, and general health (32).

The compliance was analyzed through the number of attendances of each participant at the 72 supervised lessons provided for well-tailored protocol, where 100% compliance corresponds to participation in 72 lessons, while 25% compliance corresponds to participation in 18 lessons. Compliance is expressed as a percentage of the overall lessons. Moreover, the dropout of the participants and the timing of this dropout were recorded.

### 2.3. Well-tailored training protocol

**Figure 2:**
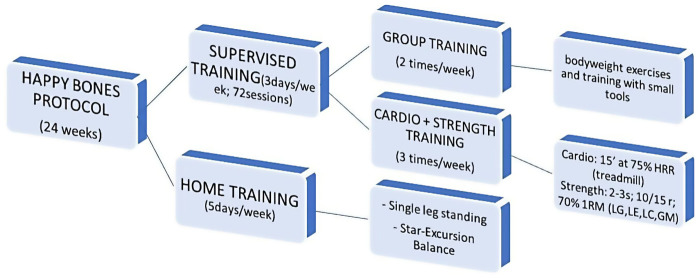
Structure of the well-tailored happy bones training protocol.

The training protocol provides 72 lessons to perform in a period of 24 weeks; it includes home training (5 days a week) and supervised training (3 days a week), performed in the working place (IW—Italy, Romania, Turkey) or sport center (SC—Bulgaria, Romania, Spain), based on the facilities available to the partners. The supervised training includes group training, strength training, and cardiovascular training, explained below (Figure 2).

#### 2.3.1. Home training

Participants are required to perform two exercises at home, the Single leg Standing, 1 min for each leg, and the Star-Excursion Balance, once a day for both legs. The aim of these exercises is to improve dynamic and static balance and stimulate the development of a new bone matrix.

#### 2.3.2. Happy bones supervised training (HB)

The supervised training is organized into three different phases: (1) group training; (2) strength training; (3) cardiovascular training, starting with a warm-up and ending with a cool-down of the major muscle groups involved in the protocol. The strength and cardiovascular training were performed 3 days per week, while the group training 2; all sessions were held under the supervision of the two specialized trainers involved in the Train the Trainer course. The group training included bodyweight exercises and training with small tools to improve balance, strengthen the trunk extensor muscles, to increase flexibility, coordination, and posture. The strength training was performed using four different machines, specific for the lower limbs: the leg press machine, the leg extension machine, the leg curl machine, and the gluteus machine. The strength session started with 2 series of 10 repetitions at 50% of the 1RM and the aim was to reach, at the end of the intervention, 2–3 series of 10/15 repetitions at 70% of the 1RM. The 1RM was calculated through the Brzycki formula mentioned above. Lastly, the cardiovascular training was performed on a treadmill or elliptical machine; it started with 15 min of approximately 75% of the heart reserve rate (HRR), evaluated through the Karvonen formula (Target Heart Rate = [(max HR−resting HR) × %Intensity] + resting HR), and at the end of the protocol women gradually achieved 30 min of activity at 75% of their HRR.

### 2.4. Statistical analysis

The statistical package IBM SPSS version 19 (IBM, Chicago, IL,USA), was used for the analysis. At first, each variable was checked for normality using the Kolmogorov-Smirnov Test. For variables that showed normal distribution, separate repeated measures with ANOVA were performed to explore the effect of time, whilst data were presented as mean values and standard deviations. In addition, effect size (ES) was calculated for all variables as partial eta-squared (h2p). Partial eta-squared values below 0.01, between 0.01 and 0.06, between 0.06 and 0.14, and above 0.14 were considered to have trivial, small, medium, and large effect sizes, respectively (33). The variables with no normal distribution were tested for pre and post-intervention differences using the non-parametric Wilcoxon signed ranked test. The median and interquartile range (1st and 3rd quartile) were chosen to represent statistical dispersion for these variables. Lastly, chi-square analysis to determine differences in compliance rates between IW vs. SC was performed. The statistical significance was set at an alpha level of *p* < 0.05.

## 3. Results

### 3.1. Results of the compliance and drop-out

This is not a Randomized Control Trial, women were enrolled in the IW or in the SC group depending on the facilities available to the partners. No significant differences between countries were reported at baseline. Out of the total 39 women IW who participated in the protocol, 5 (12.8%) had to leave the program due to problems related to the Covid-19 restrictions, as highlighted in the flowchart. Out of the 34 women who carried out the program in SC,14 of them (41.2%) did not complete the training program due to issues not related to the protocol.

Italian participants carried out the protocol at the workplace, in the university gym, 5 of them registered a high participation frequency in lessons (between 75% and 100%), 7 had a moderate attendance (between 50% and 70%), 2 registered a low frequency (between 25% and 50%) and the group had only 1 drop-out not related to the training protocol.

Bulgarian participants carried out the protocol in a sports center and were recruited through an informative video; due to the pandemic, some had to take the lessons online *via* the Zoom platform. Initially, 16 women started the program but during the six months of lessons, 9 participants left the protocol for reasons not related to the proposed physical activity. The remaining 7 women concluded the protocol by attending 100% of the lessons.

Out of the 12 Romanian participants, 6 were recruited at the university (IW) and 6 externally (SC). The group carried out the protocol in the university gym. None of the participants dropped out of the study and they all completed 100% of the proposed lessons.

Turkish participants carried out the protocol at the workplace, in the university gym. Initially, 18 women were recruited, and 14 participants concluded the exercise protocol; the other 4 left the study due to problems unrelated to the proposed training. Out of the 14 participants, 5 registered a high participation frequency in the sessions (between 75% and 100%), 7 had moderate participation (between 50% and 75%), and 2 a low participation (between 25% and 50%).

Spanish participants were recruited outside the workplace and carried out the activity in the sports center. 12 women were recruited and 5 of them had to drop out of the study due to problems that were not related to the protocol. Out of the 7 women who completed the training, 4 registered a high participation frequency in the sessions (above 75%), while 3 showed moderate compliance (between 50% and 75%).

**Figure 3:**
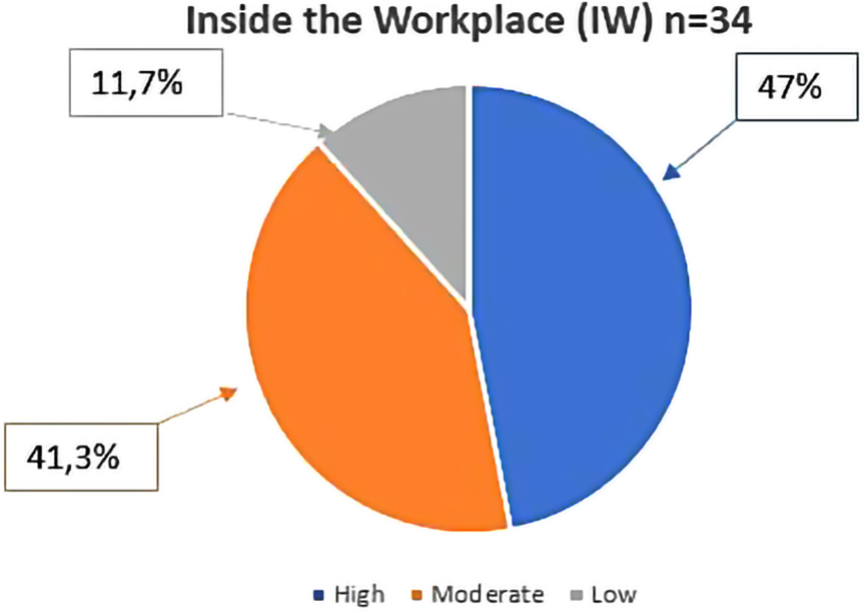
Participation rate in the protocol of women who were recruited in the working place (IW) (*n* = 34). The percentage was calculated on the total number of participants who completed the proposed activity. High = participation range between 100% and 75%; Moderate = participation range between 75% and 50%; Low = participation range between 50% and 25%.

As regards participation in the proposed training activity, out of the 34 women recruited at the workplace who completed the training, 47% recorded high compliance, 41.3% moderate compliance, and 11,7% low compliance (Figure 3).

**Figure 4:**
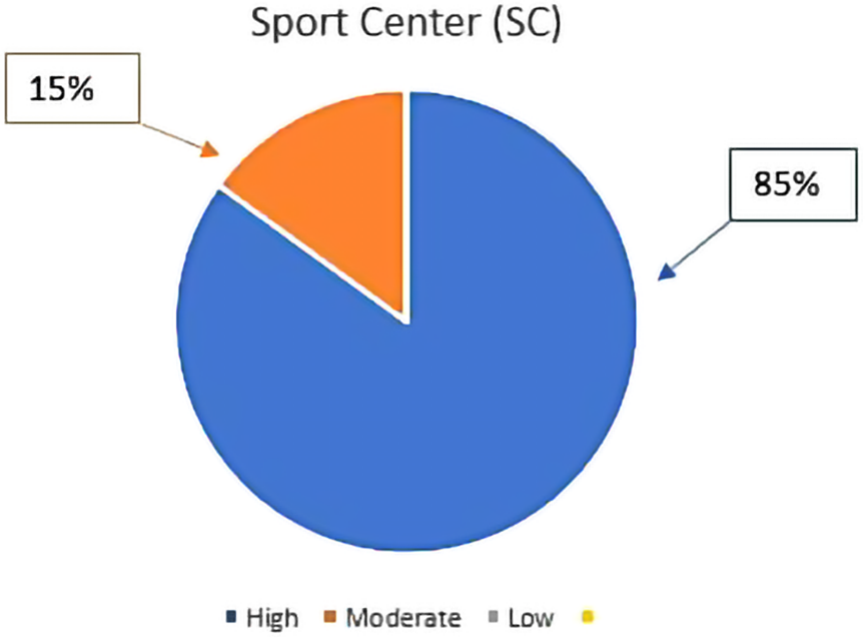
Participation rate in the protocol of women who were recruited outside the workplace (*n* = 20). The percentage was calculated on the total number of participants who completed the proposed activity. High = participation range between 100% and 75%; Moderate = participation range between 75% and 50%; Low = participation range between 50% and 25%.

Out of the 20 women recruited outside the workplace, that performed the activity in an SC, and who completed the protocol, 85% showed high participation and 15% moderate participation in the proposed training sessions (Figure 4).

The compliance to the training protocol was statistically higher in the SC compared to the IW (*χ*2(1) = 5.538; *p* = 0.019).

### 3.2. Results of the functional parameters

The functional and psychological results will be presented as a whole group because at baseline and after the protocol was no registered differences between groups (neither between nations nor IW and SC) in the parameters analyzed. At the end of the 6 months ofPA, the results of the strength and functional capacity tests showed significant improvements (Table 2). More specifically, all the evaluations made on the machines used for strength training of the lower limbs (the Leg Press, the Leg Curl, the Leg Extension, and the Gluteus Machines—*p* < 0.001) reported a statistically significant increase in this parameter. The Six minutes walking test showed a clear improvement in meters walked from about 570 before the protocol started to about 625 after its completion (*p* < 0.001). Finally, the functional test of the lower limbs (30’ sit to stand) confirmed the improvement already shown in the strength tests reported above (*p* = 0.005).

**Table 2:** Results of the total group for Pre and post functional test.

Functional Test	T0 (M ± SD)	T1 (M ± SD)	*p*-value	h2p	IC 95%
Weight (Kg)	66.9 ± 9.5	66.4 ± 9.8	n.s.	–	–
1RM LP (Kg)	69.9 ± 38.5	104.3 ± 53.4	*p* < 0.001	.581	73.86–100.41
1RM LC (Kg)	23.7 ± 14	33.5 ± 16	*p* < 0.001	.628	24.29–33.06
1RM LE (Kg)	38.4 ± 20.5	53.5 ± 20.5	*p* < 0.001	.562	40.18–51.83
1RM GM (Kg)	52.4 ± 26.3	75.1 ± 34.6	*p* < 0.001	.503	55.21–72.37
HT Right (Kg)	28.5 ± 10.7	28.6 ± 9	n. s.	–	–
HT Left (Kg)	27.8 ± 10.9	27.5 ± 8.1	n. s.	–	–
SMWT (m)	570.8 ± 103.3	625.5 ± 74	*p* < 0.001	.393	573.21–623.13
30’ StS (n)	16.3 ± 5.4	18 ± 4.3	*p* = 0.005	.164	15.83–18.54

M, mean; SD, Standard Deviation; n., numbers; 1RM, one repetition maximum; 1RM LP, 1RM Leg Press; 1RM LC, 1RM Leg Curl; 1RM LE, 1RM Leg Extension; 1RM GM, 1RM Gluteus Machine; HT, Handgrip Test; SMWT, Six Minutes Walking Test; 30’ StS, 30’ Sit to Stand; n.s., non-significative; h2p, partial eta-squared; IC, interval confidence.

All tests related to both static (Single Leg Stance—*p* = 0.001) and dynamic (Star Balance) balance showed significant improvements (Table 3). These data are very comforting as the balance is closely correlated with the risk of falls; improving this parameter can therefore prevent traumatic events that are very harmful to the general health of menopausal women who could suffer from osteopenia or osteoporosis.

**Table 3:** Results of the total group for Pre and post-functional test.

Balance Test	T0 (M ± SD)	T1 (M ± SD)	*p*-value	h2p	IC 95%
Single Leg Stance	25.5 ± 6.6	30.4 ± 8.7	*p* = 0.001	.227	26.08–29.87
Star Balance					
*Ant R*	73.4 ± 11.5	79.3 ± 12.5	*p* = 0.02	.349	70.86–79.27
*Ant Med R*	76.5 ± 11.1	83 ± 11	*p* = 0.05	.391	74.24–82.47
*Med R*	75.6 ± 12	80.6 ± 12.9	*p* = 0.05	.166	72.62–80.99
*Post Med R*	71.9 ± 13.5	81.5 ± 13.9	*p* = 0.001	.421	71.01–79.86
*Post R*	70.8 ± 12.2	82 ± 13	*p* = 0.001	.623	70.80–79.43
*Post Lat R*	67.1 ± 12.8	76.8 ± 14.2	*p* < 0.001	.544	66.37–75.14
*Lat R*	60 ± 14.2	71.2 ± 14.6	*p* < 0.001	.459	60.32–68.90
*Ant Lat R*	68.9 ± 11.2	74.7 ± 12	*p* = 0.019	.387	66.57–74.63
*Ant L*	72 ± 12	80 ± 13.8	*p* = 0.004	.524	70.31–79.12
*Ant Med L*	74.7 ± 11.1	82.2 ± 12.5	*p* = 0.003	.406	72.91–81.27
*Med L*	70.4 ± 13.1	80.2 ± 15.5	*p* = 0.001	.300	69–77–78.39
*Post Med L*	72.1 ± 10.6	80.6 ± 14.4	*p* = 0.001	.391	70.79–79.22
*Post L*	69.9 ± 13.9	82.3 ± 13.9	*p* < 0.001	.615	70.33–79.42
*Post Lat L*	68 ± 14.2	78.3 ± 12.8	*p* = 0.004	.509	67.58–76.29
*Lat L*	62.5 ± 16.4	72.7 ± 14	*p* = 0.001	.391	62.05–71.09
*Ant Lat L*	69.1 ± 11.4	76.2 ± 10.2	*p* = 0.002	.479	67.48–75.27

M, mean; SD, Standard Deviation; h2p, partial eta-squared; IC, interval confidence; ANT, anterior; MED, medial; POST, posterior; LAT, lateral; R, Right; L, Left.

The IPAQ questionnaire results showed a significant improvement in the level of activity (*p* = 0.001), which went from moderate to high, as reported by the MET which is an index linked to weekly energy expenditure due to physical activity (Table 4).

**Table 4:** Results of the total group for Pre and post IPAQ score.

IPAQ	T0 (Me-IQR)	T1 (Me-IQR)	*Z* score	Sign
MET	984.0 (1,478.0)	2,256.0 (2,160.0)	−5.090	0.001

IPAQ, international physical activity questionnaire; MET, metabolic equivalent of the task; Me, median; IQR, interquartile range; sign., significance.

### 3.3. Results of the Quality of Life Questionnaire

The Quality of Life questionnaire (Qualeffo-41), administered both before and after the training protocol implementation, showed a general improvement of this parameter in the participants (Table 5). The interpretation of this questionnaire foresees that the data that decreases detect an improvement, on the contrary, those showing an increase, detect a deterioration. The total score of the test showed a decrease of 1.2%, which indicates a general improvement in the quality of life. The scores of Mental Function (−5.4%), General Health Perception (−5.5%), Leisure, Social Activity (−0.5%), and Mobility (−2.8%) also decreased. These results, despite not being significant, suggest how the physical activity protocol led to an improvement in the participants' cognitive function, the general perception of health, sociability, and the ability to move autonomously.

**Table 5:** Results of the total group for the qualeffo-41 questionnaire in the different domains.

Intervention Group	T0 (Me-IQ)	T1 (Me-IQ)	*Z* score	Sign
Pain	30.0 (13.7–50.0)	30.0 (10.0–50.0)	−.650	n.s.
Activities of daily living	12.5 (6.2–53)	6.2 (6.2–59.3)	−.571	n.s.
Jobs Around the House	15.0 (0.0–47.5)	10.0 (0.0–62.5)	−.246	n.s.
Mobility	15.0 (3.1–50.0)	12.5 (3.1–53.1)	−.296	n.s.
Leisure, Social Activities	42.5 (15.4–16.3)	32.8 (15.4–63.3)	−.323	n.s.
General Health Perception	50.0 (33.3–66.6)	41.6 (25.0–66.6)	−.877	n.s.
Mental Function	38.8 (30.5–59.6)	36.1 (22.2–55.3)	−1.563	n.s.
Tot Qualeffo-41	27.6 (20.2–47.2)	22.6 (16.6–57.8)	−.876	n.s.

Me, median; IQ, interquartile; Qualeffo-41, quality of life for osteoporosis; Sign, significance; n.s., non-significative.

## 4. Discussion

According to our knowledge, this is one of the first studies that compared the compliance of a well-structured combined protocol proposed in two different settings to menopausal women.

### 4.1. Compliance and drop-out in HB protocol

The results show good compliance in the proposed activity with a medium-high participation rate for more than a third of the participants, both for the women enrolled inside or outside the working place. The low compliance, both in IW and SC, was affected by the covid restrictions still in place during the conduct of the protocol. This result evidence higher compliance than similar studies of PA in the workplace (34, 35). The dropout was less in the IW group concerning the SC, suggesting that a protocol proposed in the working place can reduce all those barriers that prevent a continuous practice of physical activity, i.e., lack of time or transportation. The drop-out was not related to the protocol, but due to the personal problems of the participants, regardless of the group in which they were enrolled. Throughout the study, several factors tangibly emerged that influenced, positively or negatively, the women's participation. A positive aspect was the choice to propose the protocol to employees of universities specialized in healthcare, which meant that among the participants there was greater awareness about the role of physical activity on health. Moreover, these workplaces already have well-organized facilities that can be offered to the employees. While the choice to carry out the activity during the lunch break, could represent a negative aspect, because most of the academic meetings are scheduled around this period, after the end of the lessons with students. As reported in the study of Burn et al. (36), that investigated the effectiveness of interventions of physical activity in the workplace dividing the participants into two groups “in-work” and “after-work”, the “after-work” group showed better effects and above all a higher participation rate, although in both groups there was an improvement in overall health (36). Despite this is a pilot study with a small study sample and therefore cannot lead to a generalization of the effects, compared to other studies the level of compliance IW and SC was higher, and it should encourage further studies, more extensive over time, to evaluate the long-term effects on compliance, and, in addition, it may be helpful to provide sessions during and after working hours.

### 4.2. Functional and psychological effect of HB protocol

According to the functional results, the intervention protocol proposed improved the strength of the lower limbs, as reported by the 1 RM results, as well as functional abilities, such as a 6-minute walking test and balance. Strength and balance are two fundamental components to preventing and treating osteoporosis, which occurs during the menopausal stage (37). Increasing strength allows to intervene in the maintenance of the bone and the general health of the individuals while through the improvement of balance, it is possible to work on the prevention of falls (38). Even the 30' Sit to Stand Test result highlights this significant result, evidencing an improvement in the functional lower extremity strength, which is highly correlated with the risk of falls (39). Furthermore, questionnaires were administered to all project participants to investigate the level of physical activity and quality of life. Although self-reported measurements and population physical activity levels resulting from self-assessment should be interpreted with caution (40), this qualitative analysis through the IPAQ demonstrated an improvement in the time spent actively. In terms of quality of life, the QUALEFFO-41 questionnaire did not produce statistically significant results, although the trend shows improvement. It is important to note, however, that the QUALEFFO-41 is a questionnaire primarily designed for individuals who already suffer from osteoporosis and have fractures. But since this protocol is part of a European project, involving other countries and numerous subjects, it was necessary to use this questionnaire because it is more accessible to all and its reliability and validity have been demonstrated in multiple languages (41).

### 4.3. Limitations

The results from this study were positive and very encouraging, nevertheless, the study has some limitations. Primarily this is not a randomized control trial, it was performed in 5 different countries around Europe, and for this reason, the choice to be included in the working place or in the sports center group depended on the country's possibility and not from a proper randomization. Nevertheless, due to the normal distribution of the functional and psychological data we had the possibility to evaluate the group as one and compare the data from pre to post training. Moreover, we did not analyze bone health through specific equipment such as MOC or DEXA, mainly because not all partners could reach this equipment and also because, according to the literature, a 6-month protocol is not able to produce a significant result in terms of bone health (42). In addition, to make the protocol more comprehensive and allow for good performance of the Handgrip Test as well, upper limb activities and tools for objective measurements, such as pedometers or digital step-counting watches, should have been added to the training sessions. Lastly, the QUALEFFO-41 questionnaire should have been implemented with other quality-of-life questionnaires more specific to the target population. All these elements could contribute to making further studies more in-depth and capable of producing more generalizable effects.

## 5. Conclusions

In conclusion, the Happy Bone protocol, when it is proposed in the workplace, seems to reduce the level of dropout compared to those experienced by the women that performed the training in the sports center. Moreover, the protocol improved the general health of the participants, increasing strength and balance, regardless of where it has been proposed. Given these positive and encouraging results women's national societies should collaborate with Ministries of sport, universities of sports science, national nutrition foundations/councils, nongovernmental organizations concerned with seniors' welfare, and national sports councils to inform menopausal women, and adults in general, on their nutritional and exercise needs to maintain a healthy skeleton, avoid premature bone loss, fall and avoid malnutrition in the elderly. Moreover, it would be extremely interesting to expand and extend this project over time. In addition, it would also be important to use the model of this intervention to support the prevention and management of other types of chronic diseases and populations, making the workplace one of the first places where promote health (43).

## Data Availability

The raw data supporting the conclusions of this article will be made available by the authors, without undue reservation.
